# Supplementary material for: Spa2 remodels ADP-actin via molecular condensation under glucose starvation
Source: Nat Commun. 2024 May 27;15:4491. doi: 10.1038/s41467-024-48863-4 (PMC11130202; doi:10.1038/s41467-024-48863-4)
Supplement: Supplementary file 1 — Supplementary Information [file 41467_2024_48863_MOESM1_ESM.pdf]

Supplementary information for

Spa2 remodels ADP-actin via molecular condensation under glucose starvation

Qianqian Ma[1], Wahyu Surya[1], Danxia He[1], Hanmeng Yang[1], Xiao Han[1], Mui Hoon Nai [3], Chwee Teck Lim [3,4], Jaume Torres[1], Yansong Miao [1,2*]

[*]Corresponding author. Email: yansongm@ntu.edu.sg (Y.M.)

Supplementary Figs. 1 to 7

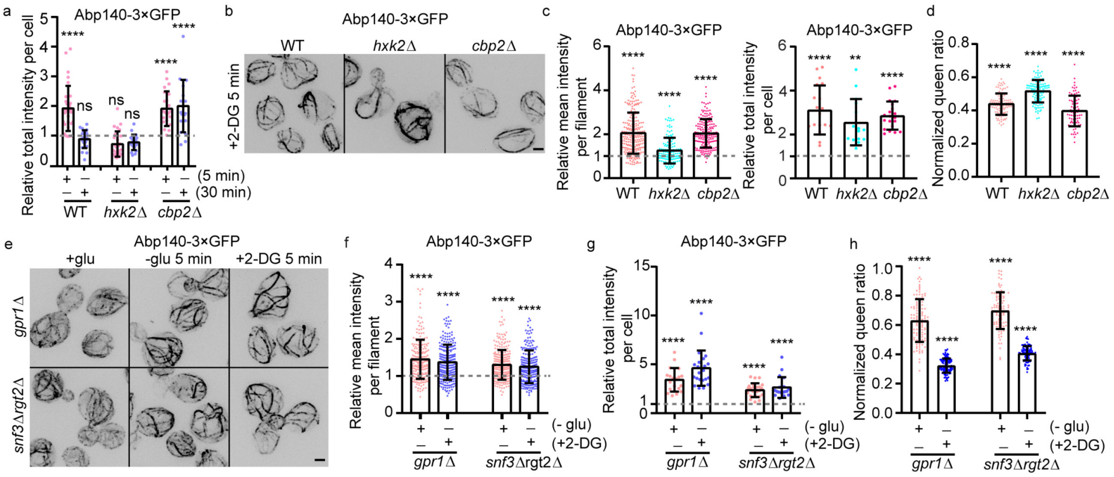

Supplementary Fig. 1. Energy starvation-induced actin cable remodeling in yeast. (**a**) Quantification of the total fluorescence intensity of the ABP140-3×GFP-labeled actin cables (n = 31, 22, 26, 20, 23, 19 cells, left to right). Dashed line indicating 1. (**b**) Representative maximum Z-projection images of ABP140-3×GFP-labeled actin cables in WT and glucose-relevant mutants *hxk2*Δ and *cbp2*Δ treated with 20 mM 2-DG for 5 min. (**c**) Quantification of the mean and total fluorescence intensity of the ABP140-3×GFP-labeled actin cables in (b) (from left to right: n = 226, 132 and 190 cables; n = 19, 15 and 20 cells). Dashed line indicating 1. (**d**) The normalized QUEEN ratio (410

nm ex/480 nm ex) in WT, *hxk2*Δ, and *cbp2*Δ upon 5 min of 2-DG treatment. The ratio was calculated from the signal intensity of each pixel to generate the QUEEN ratio image of cells (from left to right: n = 103, 105 and 79 cells). (**e**) Representative maximum Z-projection images of ABP140-3×GFP in *gpr1*Δ and *snf3*Δ*rgt2*Δ cells upon ES for 5 min and quantification of the mean (**f**) (n = 210, 247, 277 and 224 cables, left to right) or total fluorescence intensity (**g**) (n = 21, 23, 25, 19 cells, left to right). Dashed line indicating 1. (**h**) The normalized QUEEN ratio (410 nm ex/480 nm ex) in *gpr1*Δ, and *snf3*Δ*rgt2*Δ cells was calculated after 5 min of ES using the signal intensity of each pixel to generate the QUEEN ratio image of cells (from left to right: n = 115, 113, 97 and 92 cells). P-values were calculated using GraphPad Prism 6. Significance between two sets of data were determined by the one-way analysis of variance (****$p < 0.0001$). Scale bars, 2 μm.

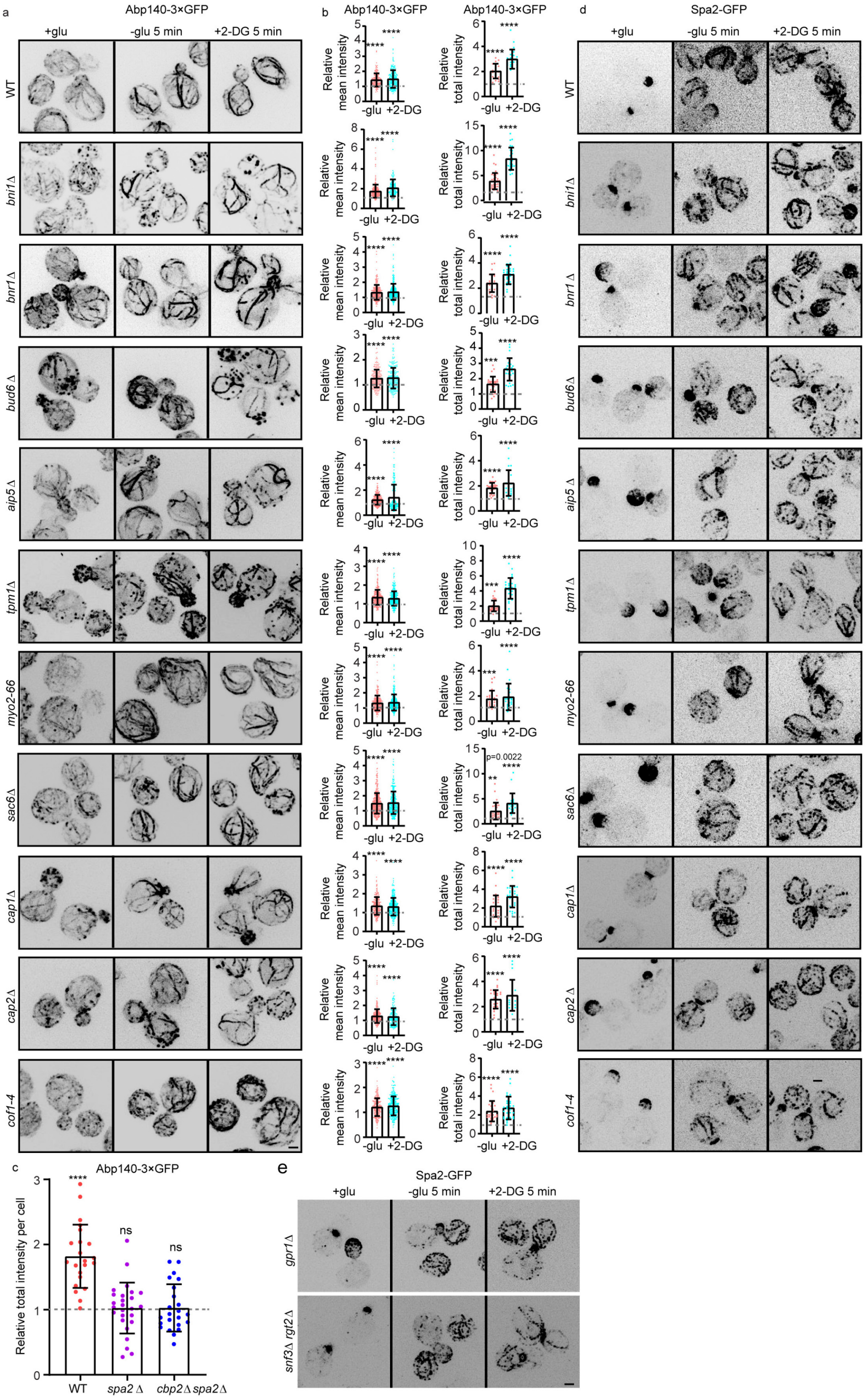

Supplementary Fig. 2. Characterization of ES-triggered actin cable changes in yeast mutants. (**a**) Representative maximum Z-projection images of ABP140-3×GFP-labeled actin cables in mutants upon 5 min ES. (**b**) Quantification of the mean and total

fluorescence intensity of the ABP140-3×GFP-labeled actin cables in (a). Dashed line indicating 1. (**c**) Quantification of the total fluorescence intensity of the ABP140-3GFP-labeled actin cables in WT, *spa2*Δ, and *spa2*Δ*cbp2*Δ cells upon 5 min of glucose starvation (n = 22, 26 and 23 cells, left to right). Dashed line indicating 1. (**d**) Representative maximum Z-projection images of Spa2-GFP in actin-binding protein mutants, or (**e**) glucose sensing mutants *gpr1*Δ *and snf3*Δ*rgt2*Δ, upon 5 min ES. P-values were calculated using GraphPad Prism 6. Significance between two sets of data were determined by the one-way analysis of variance (***p < 0.001, ****p < 0.0001, **p < 0.01 and specified and ns = not significant). Scale bar, 2 μm.

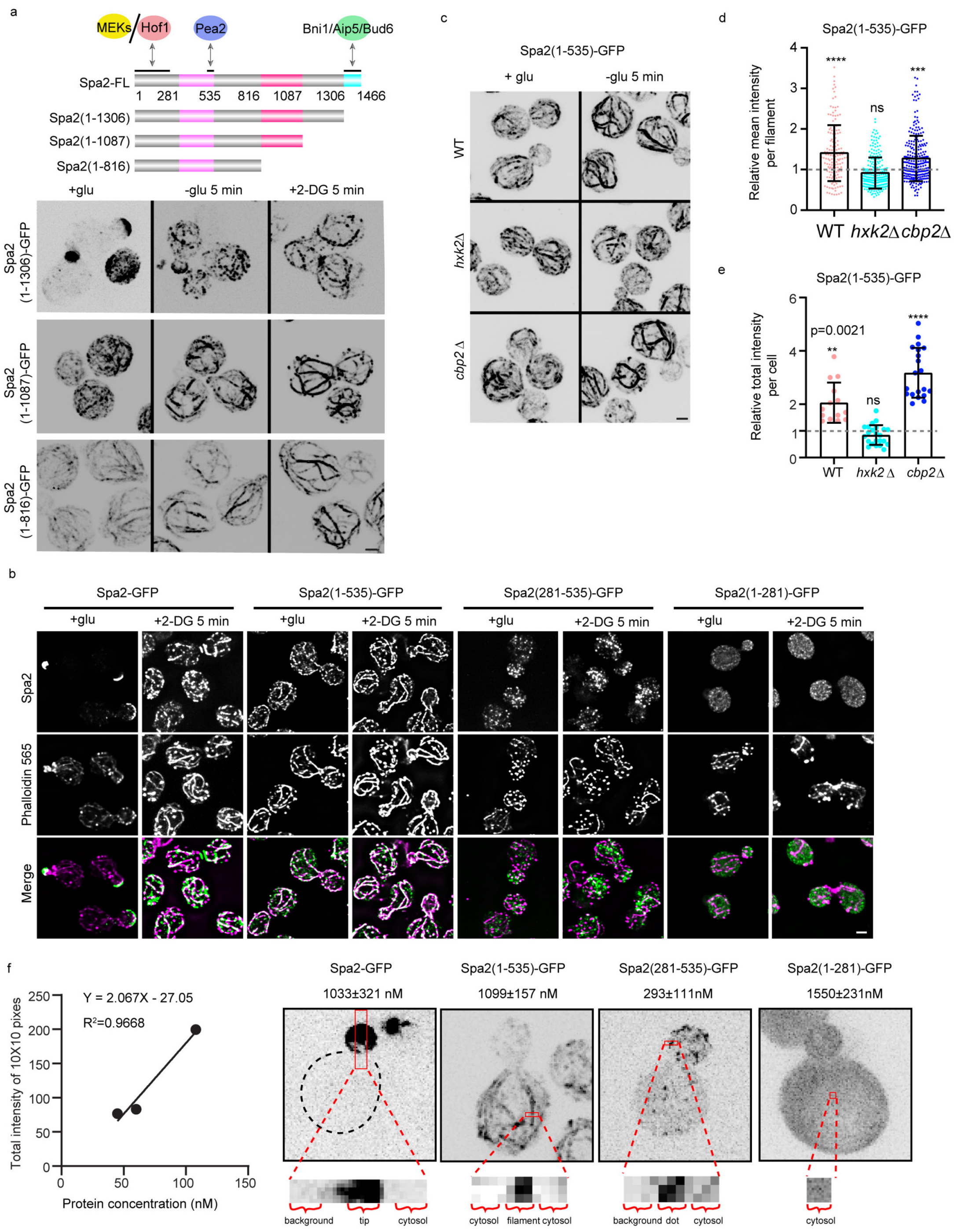

Supplementary Fig. 3. The N-terminus of Spa2-mediates actin remodeling upon ES. (**a**) Domain illustration of the Spa2 truncating variants used in the study and interacting molecules. Localization and pattern changes of GFP-tagged Spa2 truncating variants upon 5 min ES conditions. (**b**) Representative maximum Z-projection imaging of 565-phalloidin-stained yeasts expressing Spa2-GFP, Spa2(1-535)-GFP, Spa2(281-

535)-GFP, and Spa2(1-281)-GFP after 5 min of 2-DG treatment. (**c**) Representative fluorescence imaging of GFP-tagged Spa2(1-535) in the indicated mutants upon glucose starvation for 5 min. (**d**, **e**) Quantification of the mean or total signal intensity of Spa2(1-535)-GFP filaments in (c) (from left to right: n = 148, 239 and 254 per filament in (d); n = 14, 23 and 20 cells in (e). Dashed line indicating 1. Scale bar, 2 μm. (**f**) In vivo concentration of Spa2 variant proteins using signal intensity measurements, which were compared with reference proteins and the protein concentrations standard curve was generated (see Methods). The calculated Spa2 variant proteins concentrations at bud tip, filament, puncta and cytosol regions were indicated. P-values were calculated using GraphPad Prism 6. Significance between two sets of data were determined by the one-way analysis of variance (***$p < 0.001$, ****$p < 0.0001$, **$p < 0.01$ and specified and ns = not significant). The scale bar represents 2 μm.

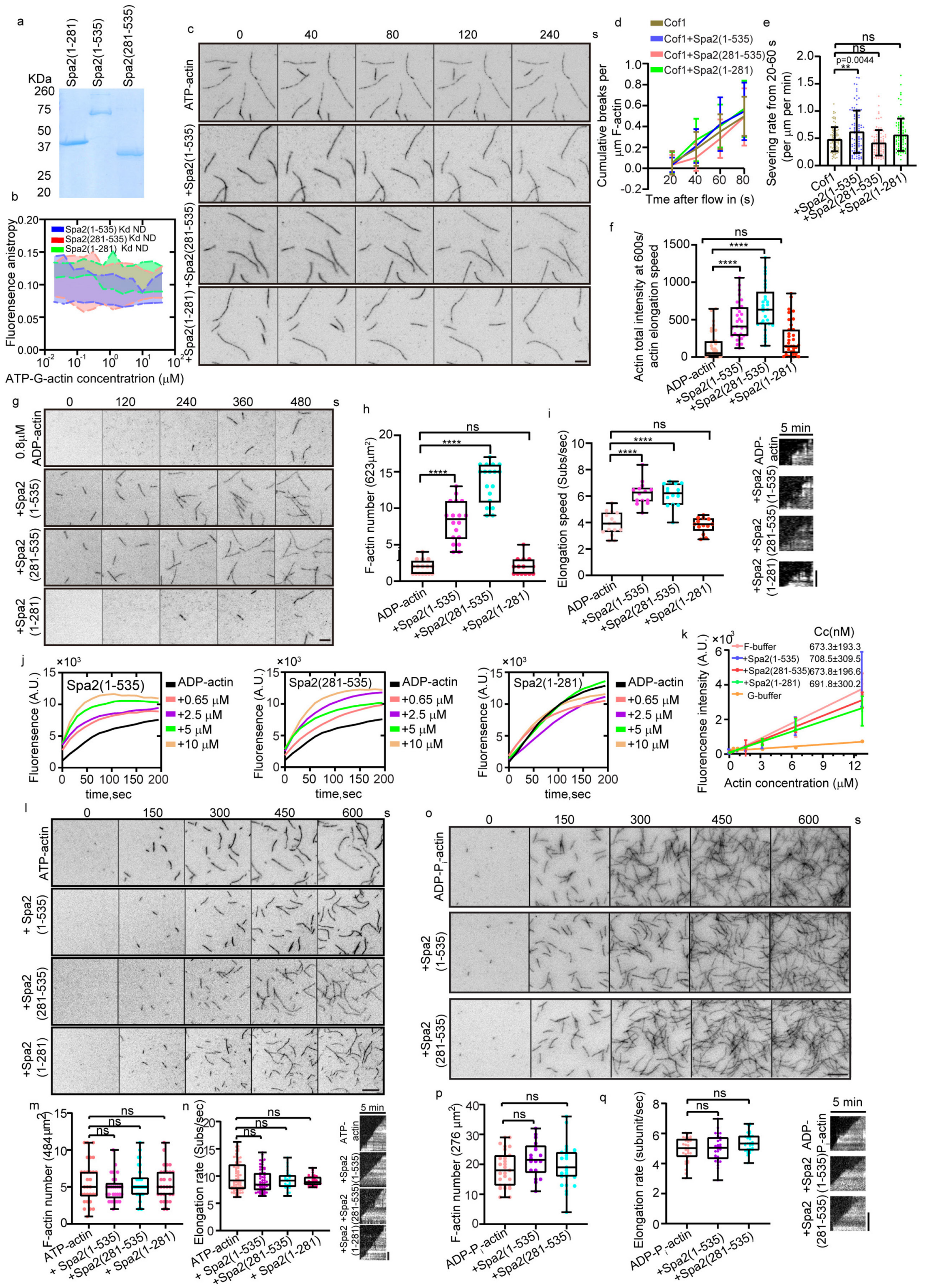

Supplementary Fig. 4. Nucleotide-specific actin polymerization by Spa2. (**a**) SDS–PAGE of bacteria purified recombinant Spa2(1-281), Spa2(1-535), and Spa2(281-535). (**b**) Anisotropic measurements of 60 nM Alexa 488-labeled Spa2-truncations titrated

with increasing concentrations of ATP-actin monomer. The average values with an error bar of ± SD were calculated from nine biological replicates and plotted with the Hill slope equation. (**c**) Representative time-lapse TIRFM images of actin filament by incubating 500 nM Spa2-truncation variants with preformed actin filament using 0.1 μM ATP-G-actin with 10% Oregon Green 488 (OG488) and 0.5% biotin labeling. (**d**) Quantification of filament cumulative breaks per micron of filament at each time point from TIRF assays. Each point averaged from 93, 98, 91, 90 filaments for Cof1, Cof1+Spa2(1-535), Cof1+Spa2(281-535), Cof1+Spa2(1-281). The cumulative breaks were analyzed by manually count the severing number on filament by ImageJ. (**e**) Quantification of maximal severing rates for each condition were determined by averaging the slopes of curves from the time interval of 20 to 60 s. Error bars, mean ± S.D. (**f**) Quantification of the ratio of total mean intensity of ADP-actin at 600s to actin filament elongation speed in Fig3d. (**g**) Representative time-lapse TIRFM images of actin polymerization at the indicated time point using 0.8 μM ADP-actin (10% OG 488- and 0.5% biotin-labeled), with or without the indicated 10 nM Spa2 variants. (**h**) Comparison of nucleation efficiency by measuring the seed number (from left to right: n = 22, 28, 29, and 29 ROIs at 24.9×24.9 μm^2). (**i**) Quantification of the actin elongation rates and kymograph in (g) (left to right, n = 34, 36, 39 and 52 actin filaments). Bars for g and i, 2 μm. Error bars, mean ± S.D. (**j**) Pyrene-ADP-actin polymerization reaction with an increased concentration of Spa2 variant truncation proteins. (**k**) Effect of Spa2 variant truncation proteins on the Cc of ADP-actin. Dependence of actin polymer concentration on total actin concentration in the absence or presence of 5 μM Spa2

variant truncation proteins. (**l**) Representative TIRFM-actin polymerization time-lapse images at the indicated time points. Actin filaments were assembled using 0.5 μM ATP-actin monomer (10% OG 488 and 0.5% biotin-labeled) with or without 10 nM Spa2(1-535), Spa2(281-535), or Spa2(1-281). The scale bar represents 5 μm. (**m**) Quantification of the number of nucleated actin seeds at 5 min (from left to right: n = 30, 29, 30 and 30, ROIs at a size of 22×22 μm^2). The box plot covers data from minimal to maximal, with the central line indicating the mean value. (**n**) Quantification of the actin elongation rates and kymograph in (l) (from left to right: n = 72, 59, 25 and 42 filaments). Data correspond to the mean ± S.D. Scale bar, 2 μm. (**o**) Representative TIRFM images of actin filaments formed at the indicated timepoint in the same field. Actin filaments were assembled using 3 μM ADP-P_i-actin (10% Oregon green 488 labeled and 0.5% biotin–actin) with or without 10 nM Spa2(1-535) and 10 nM Spa2(281-535). Scale bar, 5 μm. (**p**) Quantification of the number of nucleated actin seeds at 5 min (left to right, n = 27, 18 and 27, ROIs at a size of 16x16 μm^2). The box plot covers data from minimal to maximal, with the central line indicating the mean value. (**q**) Quantification of the actin elongation rates (mean ± S.D.) and kymograph was derived from TIRFM movies as shown in (o) in the presence of different Spa2 proteins at the concentration indicated. (left to right, n = 31, 30 and 30 actin filaments). Whiskers represent min to max in f, h, i, m, n, p, q. P-values were calculated using GraphPad Prism 6. Significance between two sets of data were determined by the one-way analysis of variance (***$p < 0.001$, ****$p < 0.0001$, **$p < 0.01$ and specified and ns = not significant). Scale bar, 2 μm.

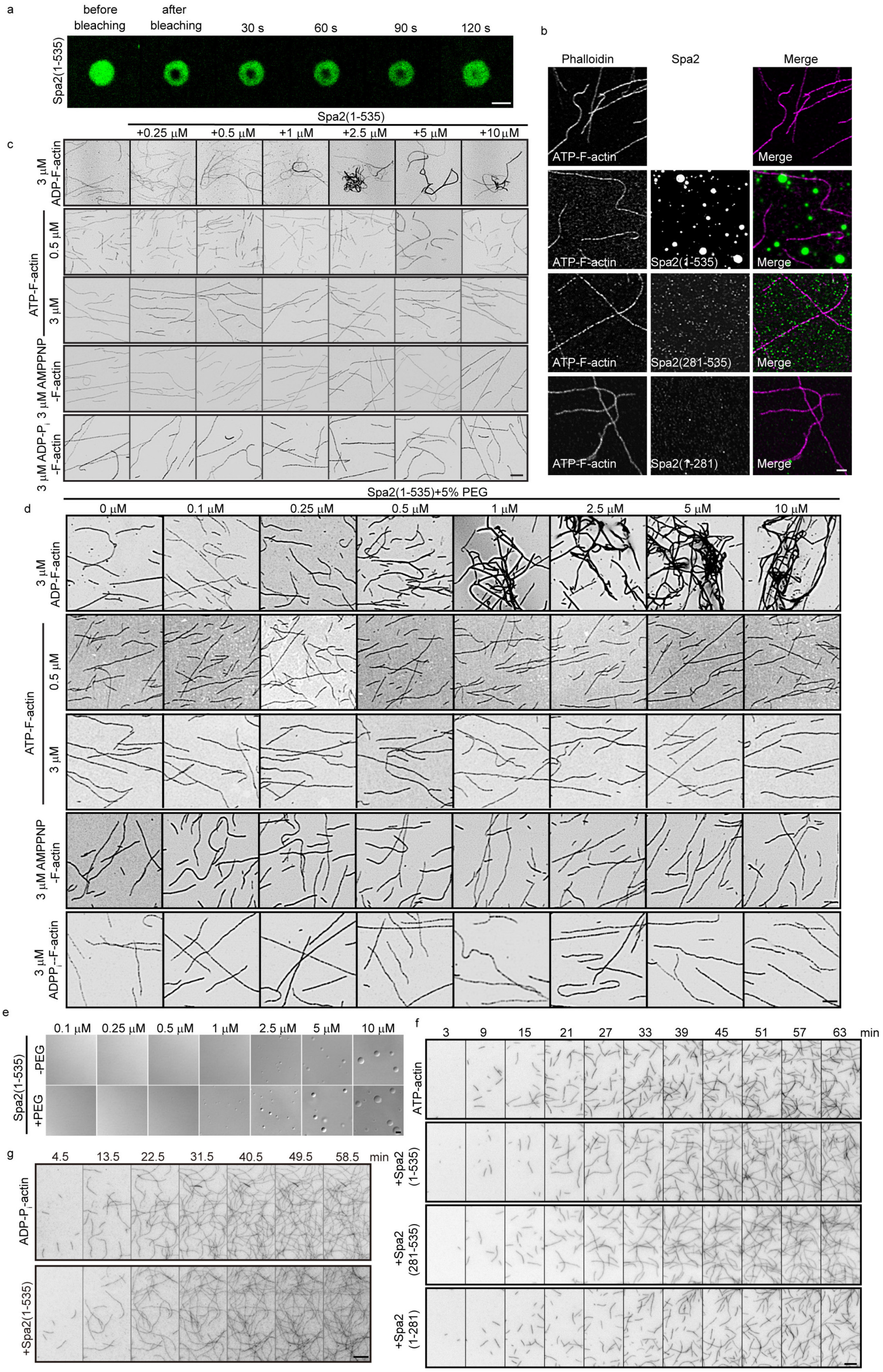
a
before bleaching
after bleaching
30 s
60 s
90 s
120 s
Spa2(1-535)
b
Phalloidin
Spa2
Merge
ATP-F-actin
Spa2(1-535)
Spa2(281-535)
Spa2(1-281)
c
Spa2(1-535)
+0.25 μM
+0.5 μM
+1 μM
+2.5 μM
+5 μM
+10 μM
3 μM ADP-F-actin
ATP-F-actin
0.5 μM
3 μM
3 μM AMPPNP -F-actin
3 μM ADP-Pi -F-actin
d
Spa2(1-535)+5% PEG
0 μM
0.1 μM
0.25 μM
0.5 μM
1 μM
2.5 μM
5 μM
10 μM
3 μM ADP-F-actin
ATP-F-actin
0.5 μM
3 μM
3 μM AMPPNP -F-actin
3 μM ADPPi--F-actin
e
0.1 μM
0.25 μM
0.5 μM
1 μM
2.5 μM
5 μM
10 μM
Spa2(1-535)
-PEG
+PEG
f
3
9
15
21
27
33
39
45
51
57
63
min
ATP-actin
+Spa2 (1-535)
+Spa2 (281-535)
+Spa2 (1-281)
g
4.5
13.5
22.5
31.5
40.5
49.5
58.5
min
ADP-Pi-actin
+Spa2(1-535)

Supplementary Fig. 5. Actin filaments are bundled by Spa2 in a nucleotide-specific manner. (**a**) Representative time-lapse images of Spa2(1-535) droplets in the FRAP assay in buffer with 50 mM KCl. (**b**) Fluorescence micrographs of actin filaments prepared from ATP-actin monomer incubated with or without 5 μM Alexa 488-labeled Spa2(1-281), Spa2(1-535), and Spa2(281-535) before being subjected to 565-phalloidin staining and imaging. (**c**) Fluorescence micrographs of actin filament prepared from ADP-actin, ATP-actin, AMPPNP-actin, and ADP-P_i-actin monomer at the indicated concentrations that were incubated with a series of concentrations of Spa2(1-535) at 0, 0.25, 0.5, 1, 2.5, 5, and 10 μM prior to 565-phalloidin staining and imaging. (**d**) Fluorescence micrographs of actin filament prepared from ADP-actin, ATP-actin, AMPPNP-actin and ADP-P_i-actin monomer at the indicated concentrations, in presence of 5% PEG, which were incubated with a series of concentrations of Spa2(1-535) at 0, 0.25, 0.5, 1, 2.5, 5, and 10 μM prior to 565-phalloidin staining and imaging. (**e**) Transmission light microscopic imaging of Spa2(1-535) phase separation at the indicated concentrations in the absence and presence of 5% PEG that were used in (d). (**f**) Representative time-lapse TIRFM images of actin filament polymerization with the indicated recombinant Spa2 variants. Actin filaments were assembled using 0.5 μM ATP-actin monomer (10% OG 488- and 0.5% biotin-labeled) with or without 5 μM Spa2(1-281), Spa2(1-535), or Spa2(281-535). (**g**) Representative time-lapse TIRFM images of ADP-P_i actin polymerization with or without the Spa2(1-535). Actin filaments were assembled using 3 μM ADP-P_i-actin (10% OG 488- and 0.5% biotin-labeled) with or without 5 μM Spa2(1-535). Scale bars are 5 μm in f, g and 2 μm in a,

b, c, d, e.

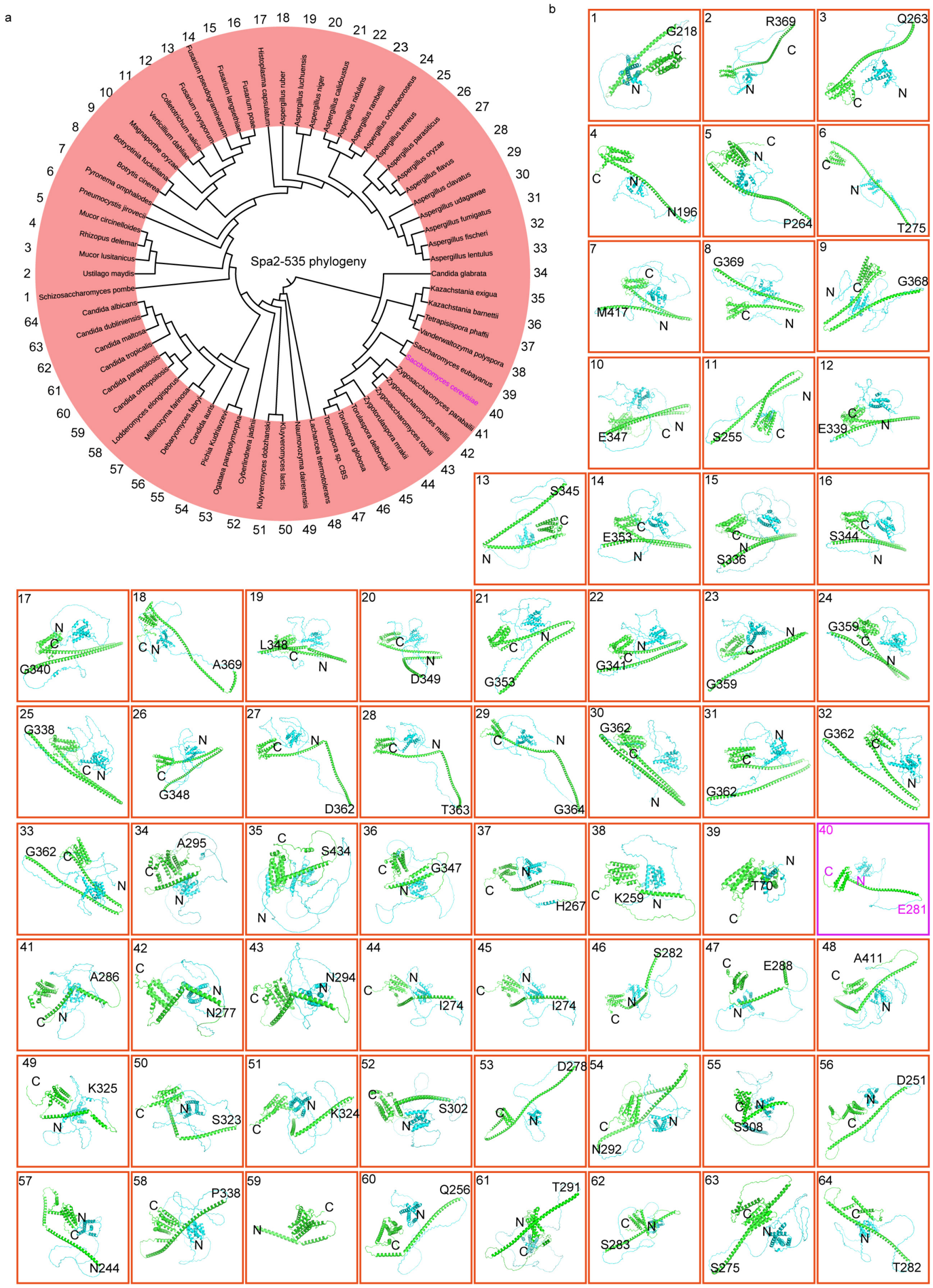

Supplementary Fig. 6. Spa2(1-535) proteins are conserved among fungi species.

(**a**) The phylogenetic tree of Spa2(1-535), the *S. cerevisiae* is highlighted. (**b**) AlphaFold prediction of Spa2(1-535) in different fungi species, the predictions of

Spa2(1-535) in *S. cerevisiae* is in magenta color.

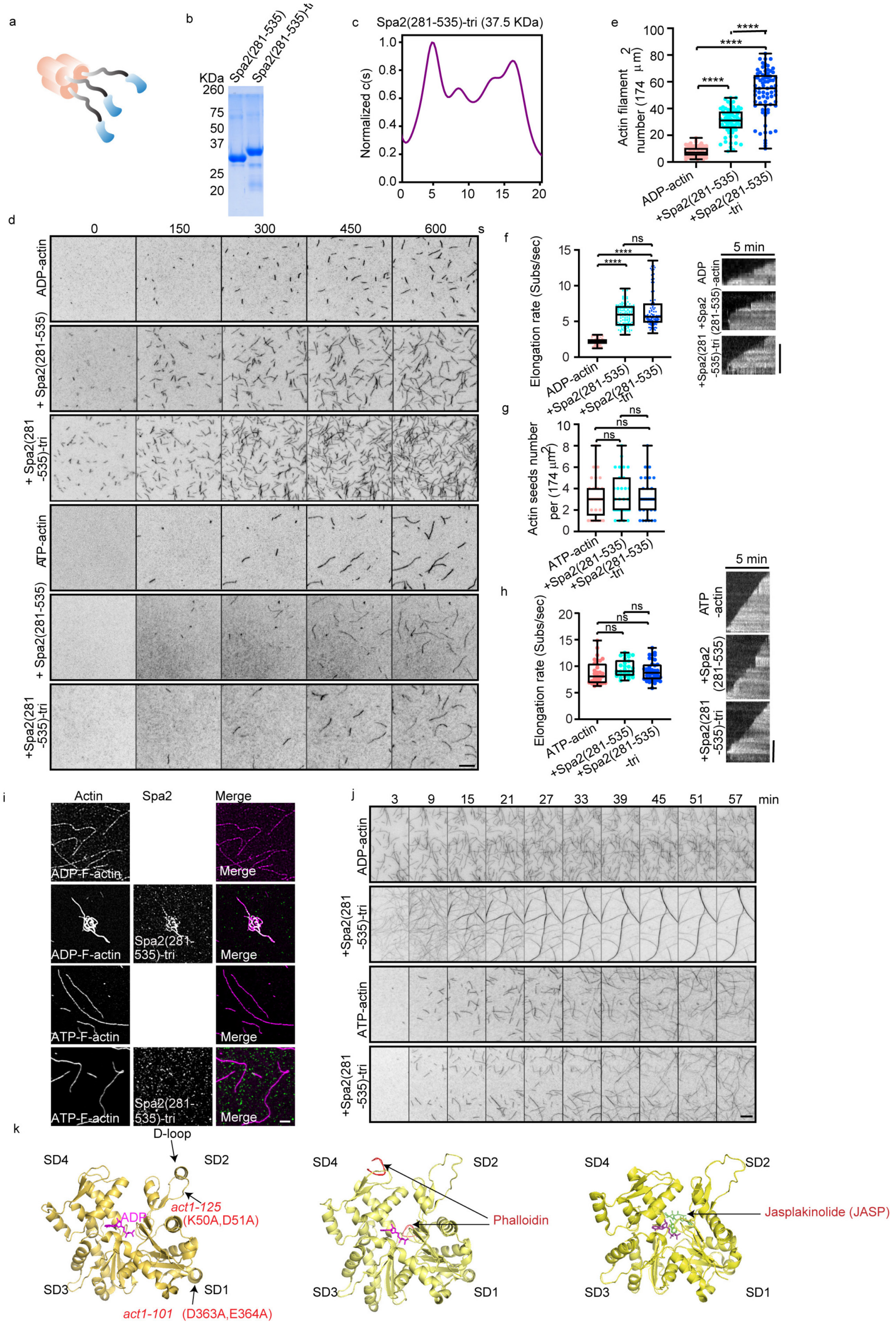

Supplementary Fig. 7. Engineered Spa2(281-535) oligomers for ADP-actin polymerization and functional characterization of D-loop for Spa2-specific

interaction. (**a**) Cartoon depicting protein engineering of Spa2(281-535) using homotrimeric CC motifs. (**b**) Comparison of Spa2(281-535) and Spa2(281-535)-trimer on SDS–PAGE. (**c**) Sedimentation velocity analysis of the Spa2(281-535)-trimer. (**d**) Representative time-lapse TIRFM images of actin polymerization. Actin filament was polymerized using 3 μM ADP-actin or 0.5 μM ATP-actin monomer (10% OG 488- and 0.5% biotin-labeled), with or without 10 nM Spa2(281-535) or Spa2(281-535)-trimer. (**e**, **f**) Quantification of nucleated ADP-actin seeds (from left to right: n = 70, 72, 72 ROIs sized at 13.2x13.2 μm^2), ADP-actin elongation rates (from left to right: n = 85, 122, 124 actin filaments) and kymograph. The box plot shows the mean ± S.D. (**g**, **h**) Quantification of ATP-actin seed generation at 8 min (from left to right: n = 29, 30, 31 ROIs at a size of 13.2x13.2 μm^2), elongation rates (from left to right: n = 33, 30, 40 actin filaments) and kymograph in the presence of 10 nM Spa2 variants. (**i**) Fluorescence micrographs of actin filaments incubated with or without 5 μM Alexa 488-labeled Spa2(281-535)-trimer prior to 565-phalloidin staining and imaging. (**j**) Representative time-lapse TIRFM images of actin filament prepared from ADP-actin or ATP-actin monomer bundling, with or without 5 μM Spa2(281-535)-trimer, using 3 μM ADP- actin or 0.5 μM ATP-actin monomer (10% OG 488- and 0.5% biotin-labeled). (**k**) Structural information of ADP-bound actin monomer (PDB code: 2hf3) with highlighted mutant sites in actin mutants *act1-125* and *act1-101*. Illustration of actin binding positions of phalloidin (PDB: 7bti) and jasplakinolide (PDB: 7ply). Whiskers represent min to max in e, f, g, h. P-values were calculated using GraphPad Prism 6. Significance between two sets of data were determined by the one-way analysis of

variance (***p < 0.001, ****p < 0.0001, and ns = not significant). Scale bars for d and j, 5 μm.

Supplementary tables

Table 1. Yeast strains used in this study.

YMY2011	MATa/MATα *his3-D200/his3-D200ura3-52/ura3-52leu2-3,112/leu2-3,112 lys2- 801/*LYS2 *ade2*-1/ADE2	Lab stock
YMY2012	*MATα his3-Δ200 leu2-3, 112 ura3-52*	Lab stock
YMY2130	MATα *his3-Δ200 leu2-3, 112, ura3-52*, Abp140-3GFP::His3	Lab stock
YMY2131	MATa *his3-Δ200 leu2-3, 112, ura3-52, spa2*Δ*::*Leu2, Abp140-3GFP::His3	Lab stock
YMY2132	MATa *his3-Δ200 leu2-3, 112, ura3-52, cbp2*Δ:: KanMX, Abp140-3GFP::His3	This study
YMY2133	MATa *his3-Δ200 leu2-3, 112, ura3-52, hxk2*Δ:: KanMX, Abp140-3GFP::His3	This study
YMY2134	MATα *his3-Δ200 leu2-3, 112, ura3-52, spa2*Δ *cbp2*Δ:: KanMX , Spa2-GFP:: His3, Abp140-Tomato:: Ura3	This study
YMY2135	MATα *his3-Δ200 leu2-3, 112, ura3-52*, Spa2-GFP:: His3, Abp140-Tomato:: Ura3	This study
YMY2136	MATa *his3-Δ200 leu2-3, 112, ura3-52*, Queen::His3	This study
YMY2137	MATa *his3-Δ200 leu2-3, 112, ura3-52*, *spa2*Δ::Leu2, Queen::His3	This study
YMY2138	MATa *his3-Δ200 leu2-3, 112, ura3-52*, *cbp2*Δ:: KanMX , Queen::His3	This study
YMY2139	MATa *his3-Δ200 leu2-3, 112, ura3-52*, *cbp2*Δ:: KanMX ,*spa2*Δ::Leu2, Queen::His3	This study
YMY2140	MATa *his3-Δ200 leu2-3, 112, ura3-52*, *hxk2*Δ:: KanMX , Queen::His3	This study
YMY2141	MATα *his3-Δ200 leu2-3, 112, ura3-52, bni1Δ::*Leu2, Abp140-3GFP::His3	This study
YMY2142	MATα *his3-Δ200 leu2-3, 112, ura3-52, bud6Δ::*Ura3, Abp140-3GFP::His3	This study
YMY2143	MATα *his3-Δ200 leu2-3, 112, ura3-52, bnr1*Δ*::*KanMX, Abp140-3GFP::His3	This study
YMY2144	MATa *his3-Δ200 leu2-3, 112, ura3-52, aip5*Δ::Ura3,	This study

	Abp140-3GFP::His3	
YMY2145	MATa *his3-Δ200 leu2-3, 112, ura3-52, tpm1*Δ::HIS3, Abp140-3GFP::His3	This study
YMY2146	MATα *his3-Δ200 leu2-3, 112, ura3-52, myo2-66::*KanMX, Abp140-3GFP::His3	This study
YMY2147	MATα *his3-Δ200 leu2-3, 112, ura3-52, sac6*Δ*::* Leu2, Abp140-3GFP::His3	This study
YMY2148	MATα *his3-Δ200 leu2-3, 112, ura3-52, cap1*Δ*::* Leu2, Abp140-3GFP::His3	This study
YMY2149	MATα *his3-Δ200 leu2-3, 112, ura3-52, cap2*Δ*::* Leu2, Abp140-3GFP::His3	This study
YMY2150	MATα *his3-Δ200 leu2-3, 112, ura3-52, cof1-4::* Leu2, Abp140-3GFP::His3	This study
YMY2151	MATa *his3-Δ200 leu2-3, 112, ura3-52, gpr1*Δ:: KanMX, Abp140-3GFP::His3	This study
YMY2152	MATa *his3-Δ200 leu2-3, 112, ura3-52*, *spa2*Δ:: KanMX, *spa2-535*:: *Leu2*, Abp140-3GFP::His3	This study
YMY2153	MATa *his3-Δ200 leu2-3, 112, ura3-52*, *spa2*Δ:: KanMX, *spa2-281-535*:: *Leu2*, Abp140-3GFP::His3	This study
YMY2154	MATa *his3-Δ200 leu2-3, 112, ura3-52*, *spa2*Δ:: KanMX, *spa2-281*:: *Leu2*, Abp140-3GFP::His3	This study
YMY2155	*MATa his3Δ1, leu2Δ0, met15Δ0, ura3Δ0, act1-101::* KanM	Lab stock
YMY2156	*MATa his3Δ1 leu2Δ0 met15Δ0 ura3Δ0, act1-159::* KanMX	Lab stock
YMY2157	*MATa his3Δ1 leu2Δ0 met15Δ0 ura3Δ0, act1-125::* KanMX	Lab stock
YMY2018	MATa *his3-Δ200 leu2-3, 112, ura3-52*, Spa2-GFP::His3	Lab stock
YMY2158	MATa *his3-Δ200 leu2-3, 112, ura3-52*, *cbp2*Δ:: KanMX, Spa2-GFP:: His3	This study
YMY2159	MATa *his3-Δ200 leu2-3, 112, ura3-52*, *hxk2*Δ:: KanMX, Spa2-GFP:: His3	This study
YMY2160	MATa *his3-Δ200 leu2-3, 112, ura3-52*, *gpr1*Δ:: KanMX, Spa2-GFP:: His3	This study
YMY2161	MATa *his3-Δ200 leu2-3, 112, ura3-52*, *snf3*Δ:: KanMX, *rgt2*Δ:: KanMX, Spa2-GFP:: His3	This study
YMY2162	MATα *his3-Δ200 leu2-3, 112, ura3-52, bni1Δ::*Leu2, Spa2-GFP:: His3	This study
YMY2163	MATα *his3-Δ200 leu2-3, 112, ura3-52, bud6Δ::*Ura3, Spa2-GFP:: His3	This study
YMY2164	MATα *his3-Δ200 leu2-3, 112, ura3-52, bnr1*Δ*::*KanMX, Spa2-GFP:: His3	This study
YMY2165	MATa *his3-Δ200 leu2-3, 112, ura3-52*, aip5Δ::Ura3,	This study

	Spa2-GFP:: His3	
YMY2166	MATa *his3-Δ200 leu2-3, 112, ura3-52*, tpm1Δ::HIS3, Spa2-GFP:: His3	This study
YMY2167	MATα *his3-Δ200 leu2-3, 112, ura3-52, myo2-66::*KanMX, Spa2-GFP:: His3	This study
YMY2168	MATα *his3-Δ200 leu2-3, 112, ura3-52, sac6*Δ*::* Leu2, Spa2-GFP:: His3	This study
YMY2169	MATα *his3-Δ200 leu2-3, 112, ura3-52, cap1*Δ*::* Leu2, Spa2-GFP:: His3	This study
YMY2170	MATα *his3-Δ200 leu2-3, 112, ura3-52, cap2*Δ*::* Leu2, Spa2-GFP:: His3	This study
YMY2171	MATα *his3-Δ200 leu2-3, 112, ura3-52, cof1-4::* Leu2, Spa2-GFP:: His3	This study
YMY2172	MATa *his3-Δ200 leu2-3, 112, ura3-52*, Spa2-535-GFP::His3	This study
YMY2173	MATa *his3-Δ200 leu2-3, 112, ura3-52*, Spa2-281-GFP::His3	This study
YMY2174	MATa *his3-Δ200 leu2-3, 112, ura3-52*, *spa2*Δ*::*Leu2, Spa2-281-535-GFP:: Ura3	This study
YMY2175	MATa *his3-Δ200 leu2-3, 112, ura3-52*, Spa2-1306-GFP:: His3	This study
YMY2176	MATa *his3-Δ200 leu2-3, 112, ura3-52*, Spa2-1087-GFP:: His3	This study
YMY2177	MATa *his3-Δ200 leu2-3, 112, ura3-52*, Spa2-816-GFP:: His3	This study
YMY2178	MATa *his3-Δ200 leu2-3, 112, ura3-52*, *cbp2*Δ:: KanMX, Spa2-535-GFP::His3	This study
YMY2179	MATa *his3-Δ200 leu2-3, 112, ura3-52*, *hxk2*Δ:: KanMX, Spa2-535-GFP::His3	This study

Table 2. Primers used in this study.

Spa2-535-GFP-F	CGAAAATAAAATCCGATTCAAATGGTGAGAGCAC CACCTCGGATCCCCGGGTTAATTAA
Spa2-535-GFP-R	CTTTGTCTTCCTTTTCTTTCTCCTCTAGATACTACT AACTGAATTCGAGCTCGTTTAAAC
Spa2-281-GFP-F	TCCCGAACAGTTGAAGAGCCCTGAAGTACAACG GGCTGAGCGGATCCCCGGGTTAATTAA
Spa2-816-GFP-F	AACTACCTGCAAATATAGTGGAACTTGATTTACAT GAGCGGATCCCCGGGTTAATTAA
Spa2-1087-GFP-F	CCATCTTCAGCTACACTGAAAAAGAGCGGGCTCC CACGGATCCCCGGGTTAATTAA
PRS306-Spa2-pro-F	GCGGTGGCGGCCGCTCTAGAGTATACATATAATCA CGTAA
PRS306-Spa2-pro-R	GTTTACTCTTTCTTTTCTTTTGTTTATTCTGTTTCG GTGG
PRS306 Spa2-281-535-F	AAAGAAAAGAAAGAGTAAACGAGAACAATA ACCCTAACTC
PRS306-Spa2-281-535-R	GGATCCGTCGACCTGCAGCGGAGGTGGTGCTCTC ACCATT
PRS306-GFP-F	ATTCAAATGGTGAGAGCACCACCTCCGCTGCAGG TCGAC
PRS306-GFP-R	GAGTTAGGGTTATTGTTCTCGTTTACTCTT TCTTTTCTTT
