# Supplementary material for: Spa2 remodels ADP-actin via molecular condensation under glucose starvation
Source: Nat Commun. 2024 May 27;15:4491. doi: 10.1038/s41467-024-48863-4 (PMC11130202; doi:10.1038/s41467-024-48863-4)
Supplement: Supplementary file 25 — Reporting Summary [file 41467_2024_48863_MOESM25_ESM.pdf]

nature portfolio

Corresponding author(s): Yansong Miao

Last updated by author(s): Mar 4, 2024

Reporting Summary

Nature Portfolio wishes to improve the reproducibility of the work that we publish. This form provides structure for consistency and transparency in reporting. For further information on Nature Portfolio policies, see our Editorial Policies and the Editorial Policy Checklist.

Please do not complete any field with "not applicable" or n/a. Refer to the help text for what text to use if an item is not relevant to your study.
For final submission: please carefully check your responses for accuracy; you will not be able to make changes later.

Statistics

For all statistical analyses, confirm that the following items are present in the figure legend, table legend, main text, or Methods section.

n/a	Confirmed	
☐	☒	The exact sample size (n) for each experimental group/condition, given as a discrete number and unit of measurement
☐	☒	A statement on whether measurements were taken from distinct samples or whether the same sample was measured repeatedly
☐	☒	The statistical test(s) used AND whether they are one- or two-sided<br>*Only common tests should be described solely by name; describe more complex techniques in the Methods section.*
☐	☒	A description of all covariates tested
☐	☒	A description of any assumptions or corrections, such as tests of normality and adjustment for multiple comparisons
☐	☒	A full description of the statistical parameters including central tendency (e.g. means) or other basic estimates (e.g. regression coefficient) AND variation (e.g. standard deviation) or associated estimates of uncertainty (e.g. confidence intervals)
☐	☒	For null hypothesis testing, the test statistic (e.g. F, t, r) with confidence intervals, effect sizes, degrees of freedom and P value noted<br>*Give P values as exact values whenever suitable.*
☒	☐	For Bayesian analysis, information on the choice of priors and Markov chain Monte Carlo settings
☒	☐	For hierarchical and complex designs, identification of the appropriate level for tests and full reporting of outcomes
☒	☐	Estimates of effect sizes (e.g. Cohen's d, Pearson's r), indicating how they were calculated

Our web collection on statistics for biologists contains articles on many of the points above.

Software and code

Policy information about availability of computer code

Data collection	SPR binding data were collected using Biacore T200 Evaluation software (GE Healthcare).<br>Protein sequences were obtained from FungiDB (https://fungidb.org/fungidb/) and NCBI.<br>Fluorescence images were aquired by Metamorph.<br>AFM data was aquired by Bruker Dimension FastScan AFM system and Nanoscope V controller operating under ScanAsyst fluid mode.<br>Actin anisotropy data was collected by plate reader Cytation 5 (BioTek, USA).
Data analysis	As detailed in the materials and methods, AUC data were analyzed using SEDFIT using c(s) and c(s, ff0) size distribution models and plotted with GUSSI.<br>AFM data were analyzed using Bruker Nanoscope Analysis 1.90 software.<br>Structures of the ScSpa2-535 homologs in other fungi species were predicted by Alphafold2.<br>All statistical analyses were performed in GraphPad Prism 6.<br>Protein sequence alignment was performed with the online server Clustal Omega (https://www.ebi.ac.uk/Tools/msa/clustalo/), and the phylogenetic tree was generated by the interactive tree of life (http://itol.embl.de/).<br>ANCHOR (http://anchor.enzim.hu/) and the PHYRE2 protein fold recognition server (http://www.sbg.bio.ic.ac.uk/~phyre/) were used.<br>Fluorescence images were analyzed by Fiji.

For manuscripts utilizing custom algorithms or software that are central to the research but not yet described in published literature, software must be made available to editors and reviewers. We strongly encourage code deposition in a community repository (e.g. GitHub). See the Nature Portfolio guidelines for submitting code & software for further information.

Data

Policy information about availability of data

All manuscripts must include a data availability statement. This statement should provide the following information, where applicable:

- Accession codes, unique identifiers, or web links for publicly available datasets
- A description of any restrictions on data availability
- For clinical datasets or third party data, please ensure that the statement adheres to our policy

Source data are provided as a Source Data file. The datasets generated and analysed during the current study are available in the figshare repository (https://figshare.com/s/f1d73eb1ff79bdd92615).

Research involving human participants, their data, or biological material

Policy information about studies with human participants or human data. See also policy information about sex, gender (identity/presentation), and sexual orientation and race, ethnicity and racism.

Reporting on sex and gender	No research on humans was done.
Reporting on race, ethnicity, or other socially relevant groupings	No research on humans was done.
Population characteristics	No research on humans was done.
Recruitment	No research on humans was done.
Ethics oversight	No research on humans was done.

Note that full information on the approval of the study protocol must also be provided in the manuscript.

Field-specific reporting

Please select the one below that is the best fit for your research. If you are not sure, read the appropriate sections before making your selection.

☒ Life sciences ☐ Behavioural & social sciences ☐ Ecological, evolutionary & environmental sciences

Life sciences study design

All studies must disclose on these points even when the disclosure is negative.

Sample size	No sample size calculation were performed. All the sample size was determined based on experiments, literature, and enough number for sufficient statistical analysis.
Data exclusions	no data was excluded from the analysis
Replication	Cell imaging experiments were performed at least in three independent replicates. All biochemical experiments have at least two biological replicates.
Randomization	ROIs were randomly chosen.
Blinding	Single blind analysis was applied to the imaging processing and analyzing

Reporting for specific materials, systems and methods

We require information from authors about some types of materials, experimental systems and methods used in many studies. Here, indicate whether each material, system or method listed is relevant to your study. If you are not sure if a list item applies to your research, read the appropriate section before selecting a response.

Materials & experimental systems

n/a	Involved in the study	
☒	☐	Antibodies
☐	☒	Eukaryotic cell lines
☒	☐	Palaeontology and archaeology
☒	☐	Animals and other organisms
☒	☐	Clinical data
☒	☐	Dual use research of concern
☒	☐	Plants

Methods

n/a	Involved in the study	
☒	☐	ChIP-seq
☒	☐	Flow cytometry
☒	☐	MRI-based neuroimaging

Eukaryotic cell lines

Policy information about cell lines and Sex and Gender in Research

Cell line source(s)	Sf9 cell line from ThermoFisher.
Authentication	The cell line was not authenticated prior to use.
Mycoplasma contamination	The cell line was not tested for Mycoplasma prior to use.
Commonly misidentified lines (See ICLAC register)	No commonly misidentified cell lines were used in this study.

Plants

Seed stocks	*Report on the source of all seed stocks or other plant material used. If applicable, state the seed stock centre and catalogue number. If plant specimens were collected from the field, describe the collection location, date and sampling procedures.*
Novel plant genotypes	*Describe the methods by which all novel plant genotypes were produced. This includes those generated by transgenic approaches, gene editing, chemical/radiation-based mutagenesis and hybridization. For transgenic lines, describe the transformation method, the number of independent lines analyzed and the generation upon which experiments were performed. For gene-edited lines, describe the editor used, the endogenous sequence targeted for editing, the targeting guide RNA sequence (if applicable) and how the editor was applied.*
Authentication	*Describe any authentication procedures for each seed stock used or novel genotype generated. Describe any experiments used to assess the effect of a mutation and, where applicable, how potential secondary effects (e.g. second site T-DNA insertions, mosiacism, off-target gene editing) were examined.*

This checklist template is licensed under a Creative Commons Attribution 4.0 International License, which permits use, sharing, adaptation, distribution and reproduction in any medium or format, as long as you give appropriate credit to the original author(s) and the source, provide a link to the Creative Commons license, and indicate if changes were made. The images or other third party material in this article are included in the article's Creative Commons license, unless indicated otherwise in a credit line to the material. If material is not included in the article's Creative Commons license and your intended use is not permitted by statutory regulation or exceeds the permitted use, you will need to obtain permission directly from the copyright holder. To view a copy of this license, visit http://creativecommons.org/licenses/by/4.0/
