# Supplementary material for: Spa2 remodels ADP-actin via molecular condensation under glucose starvation
Source: Nat Commun. 2024 May 27;15:4491. doi: 10.1038/s41467-024-48863-4 (PMC11130202; doi:10.1038/s41467-024-48863-4)
Supplement: Supplementary file 27 — Description of Additional Supplementary Files [file 41467_2024_48863_MOESM27_ESM.pdf]

Title: Supplementary Movie 1.

Description: Time lapse movie of ABP140-3×GFP labeled actin cable remodeling response to GS.

Time-lapse movie of ABP140-3×GFP labeled actin cable remodeling in WT, *hxk2*Δ, and *cbp2*Δ cells upon glucose starvation from 0 to 30 min. Scale bar, 2 μm.

Title: Supplementary Movie 2.

Description: Time lapse movie of the ATP sensor Queen upon to GS.

Time-lapse movie of Quenn upon glucose starvation from 0 to 30 min. Scale bar, 2 μm.

Title: Supplementary Movie 3.

Description: Time lapse movie of ABP140-3×GFP labeled actin cable remodeling response to GS 5 min.

Time-lapse movie of ABP140-3×GFP labeled actin cable remodeling in WT, *spa2*Δ, and *cbp2*Δ *spa2*Δ cells upon glucose starvation from 0 to 5 min. Scale bar, 2 μm.

Title: Supplementary Movie 4.

Description: Time lapse movie of Spa2-GFP, ABP140-Tomato response to GS 5 min.

Time-lapse movie of Spa2-GFP, ABP140-Tomato upon glucose starvation from 0 to 5 min. Scale bar, 2 μm.

Title: Supplementary Movie 5.

Description: Time lapse movie of Spa2-GFP, ABP140-Tomato response to 2-DG 5 min.

Time-lapse movie of Spa2-GFP, ABP140-Tomato upon 2-DG from 0 to 5 min. Scale bar, 2 μm.

Title: Supplementary Movie 6.

Description: Time lapse movie of Spa2-GFP response to GS.

Time-lapse movie of Spa2-GFP in WT, *hxk2*Δ, and *cbp2*Δ cells upon glucose starvation from 0 to 30 min. Scale bar, 2 μm.

Title: Supplementary Movie 7.

Description: Time lapse movie of GFP tagged Spa2 truncation variants response to GS 5min.

Time-lapse movie of Spa2(1-535)-GFP, Spa2(281-535)-GFP, Spa2(1-281)-GFP cells upon glucose starvation from 0 to 5 min. Scale bar, 2 μm.

Title: Supplementary Movie 8.

Description: Time lapse movie of GFP tagged Spa2 truncation variants response to 2-DG 5min.

Time-lapse movie of Spa2(1-535)-GFP, Spa2(281-535)-GFP, Spa2(1-281)-GFP cells upon energy starvation from 0 to 5 min. Scale bar, 2 μm.

Title: Supplementary Movie 9.

Description: Time lapse movie of ABP140-3×GFP labeled actin cable remodeling in Spa2 truncation variant response to GS 5 min.

Time-lapse movie of ABP140-3×GFP labeled actin cable remodeling in *spa2(1-535)*, *spa2(281-535)*, *spa2(1-281)* cells upon glucose starvation from 0 to 5 min. Scale bar, 2 μm.

Title: Supplementary Movie 10.

Description: Time lapse movie of ABP140-3×GFP labeled actin cable remodeling in Spa2 truncation variant response to 2-DG 5 min.

Time-lapse movie of ABP140-3×GFP labeled actin cable remodeling in *spa2(1-535)*, *spa2(281-535)*, *spa2(1-281)* cells upon 2-DG from 0 to 5 min. Scale bar, 2 μm.

Title: Supplementary Movie 11.

Description: Time lapse movie of Actin filament severing by 50nM Cof1 with or without 500 nM Spa2 variants.

TIRF movie of actin severing in the presence of indicated Cof1 with or without Spa2 variant within a time range between 0 to 10 min. Reaction contains 0.1 μM actin, 50 nM Cof1, 500 nM Spa2 variants. Scale bar, 2 μm.

Title: Supplementary Movie 12.

Description: Time-lapse movie of 3 μM ADP-actin actin polymerization by 10 nM of each Spa2 variant.

TIRF movie of Spa2 variants-mediated ADP-actin polymerization within a time range between 0 to 10 min. Reaction contains 3 μM ADP-actin, 10 nM Spa2 protein variants. Scale bar, 2 μm.

Title: Supplementary Movie 13.

Description: Time-lapse movie of 0.5 μM ATP-actin polymerization by 10 nM of each Spa2 variant.

TIRF movie of Spa2 variants-mediated ATP-actin polymerization within a time range between 0 to 10 min. Reaction contains 0.5 μM ATP-actin, 10 nM Spa2 protein variants. Scale bar, 2 μm.

Title: Supplementary Movie 14.

Description: Time-lapse movie of 3 μM ADP-P_i-actin polymerization by 10 nM of each Spa2 variant.

TIRF movie of Spa2 variants-mediated ADP-P_i-actin polymerization within a time range between 0 to 10 min. Reaction contains 3 μM ADP-P_i-actin, 10 nM Spa2 protein variants. Scale bar, 2 μm.

Title: Supplementary Movie 15.

Description: Transmission light time-lapse movie of the coalescence of 5 μM Spa2-535.

Time-lapse movie of 5 μM Spa2-535 phase separation droplet dynamics in 20 mM HEPES, 50 mM KCl pH 7.4 buffer. Scale bar, 5 μm.

Title: Supplementary Movie 16.

Description: Time lapse movie of 3 μM ADP-actin polymerization and bundling in the presence of 5 μM of Spa2 variants.

TIRF movie of Spa2 variants-mediated ADP-actin polymerization within a time range between 3 to 61.5 min. Reaction contains 3 μM ADP-actin, 5 μM Spa2 protein variants. Scale bar, 2 μm.

Title: Supplementary Movie 17.

Description: Time lapse movie of 3 μM ADP-actin polymerization without or with 5 μM Spa2-535 and then severing by 100nM Cof1 .

TIRF movie of Spa2-535 mediated ADP-actin bundles severing by Cof1. Reaction contains 3 μM ADP-actin, 5 μM Spa2-535, 100nM Cof1. Scale bar, 2 μm.

Title: Supplementary Movie 18.

Description: Time lapse movie of 0.5 μM ATP-actin polymerization in the presence of 5 μM Spa2 variants.

TIRF movie of Spa2 variants-mediated ATP-actin polymerization within a time range between 0 to 60 min. Reaction contains 0.5 μM ATP-actin, 5 μM Spa2 protein variants. Scale bar, 2 μm.

Title: Supplementary Movie 19.

Description: Time lapse movie of 3 μM ADP-P_i-actin polymerization with 5 μM Spa2-535.

TIRF movie of Spa2-535-mediated ADP-P_i-actin polymerization within a time range between 4.5 to 58.5 min. Reaction contains 3 μM ADP-P_i-actin, 5 μM Spa2-535. Scale bar, 2 μm.

Title: Supplementary Movie 20.

Description: Time lapse movie of 3 μM ADP-actin polymerization by engineered 10 nM Spa2-281-535-trimer.

TIRF movie of Spa2 variants-mediated ADP-actin polymerization within a time range between 0 to 10 min. Reaction contains 3 μM ADP-actin, 10 nM Spa2 protein variants. Scale bar, 2 μm.

Title: Supplementary Movie 21.

Description: Time lapse movie of 3 μM ADP-actin polymerization and bundling in the presence of 5 μM of Spa2-281-535-trimer.

TIRF movie of Spa2-281-535-trimer-mediated ADP-actin polymerization within a time range between 0 to 60 min. Reaction contains 3 μM ADP-actin, 5 μM Spa2-281-535-trimer. Scale bar, 2 μm.

Title: Supplementary Movie 22.

Description: Time lapse movie of 0.5 μM ATP-actin polymerization by engineered 10 nM Spa2-281-535-trimer.

TIRF movie of Spa2 variants-mediated ATP-actin polymerization within a time range between 0 to 10 min. Reaction contains 0.5 μM ATP-actin, 10 nM Spa2 protein variants. Scale bar, 2 μm.

Title: Supplementary Movie 23.

Description: Time lapse movie of 0.5 μM ATP-actin polymerization and bundling in the presence of 5 μM of Spa2-281-535-trimer.

TIRF movie of Spa2-281-535-trimer-mediated ATP-actin polymerization within a time range between 0 to 60 min. Reaction contains 0.5 μM ATP-actin, 5 μM Spa2-281-535-trimer. Scale bar, 2 μm.
